# Similar Yet Different–Structural and Functional Diversity among *Arabidopsis thaliana* LEA_4 Proteins

**DOI:** 10.3390/ijms21082794

**Published:** 2020-04-17

**Authors:** Patrick Knox-Brown, Tobias Rindfleisch, Anne Günther, Kim Balow, Anne Bremer, Dirk Walther, Markus S. Miettinen, Dirk K. Hincha, Anja Thalhammer

**Affiliations:** 1 Physical Biochemistry, University of Potsdam, Karl-Liebknecht-Str. 24–25, D-14476 Potsdam, Germany; knoxbrown@uni-potsdam.de (P.K.-B.); rindflei@uni-potsdam.de (T.R.); 2 Max-Planck Institute of Molecular Plant Physiology, Am Mühlenberg 1, D-14476 Potsdam, Germany; anne2408@gmx.net (A.G.); kimsab@mailbox.org (K.B.); Anne.Bremer@STJUDE.ORG (A.B.); Walther@mpimp-golm.mpg.de (D.W.); Hincha@mpimp-golm.mpg.de (D.K.H.); 3 Department for Structural Biology, St. Jude Children’s Research Hospital, Memphis, TN 38105, USA; 4 Max-Planck Institute of Colloids and Interfaces, Am Mühlenberg 1, D-14476 Potsdam, Germany; Markus.Miettinen@mpikg.mpg.de

**Keywords:** IDP, LEA protein, abiotic stress, dehydration, conformational rearrangement, membrane stabilization, sequence-structure-function relationship

## Abstract

The importance of intrinsically disordered late embryogenesis abundant (LEA) proteins in the tolerance to abiotic stresses involving cellular dehydration is undisputed. While structural transitions of LEA proteins in response to changes in water availability are commonly observed and several molecular functions have been suggested, a systematic, comprehensive and comparative study of possible underlying sequence-structure-function relationships is still lacking. We performed molecular dynamics (MD) simulations as well as spectroscopic and light scattering experiments to characterize six members of two distinct, lowly homologous clades of LEA_4 family proteins from *Arabidopsis thaliana*. We compared structural and functional characteristics to elucidate to what degree structure and function are encoded in LEA protein sequences and complemented these findings with physicochemical properties identified in a systematic bioinformatics study of the entire *Arabidopsis thaliana* LEA_4 family. Our results demonstrate that although the six experimentally characterized LEA_4 proteins have similar structural and functional characteristics, differences concerning their folding propensity and membrane stabilization capacity during a freeze/thaw cycle are obvious. These differences cannot be easily attributed to sequence conservation, simple physicochemical characteristics or the abundance of sequence motifs. Moreover, the folding propensity does not appear to be correlated with membrane stabilization capacity. Therefore, the refinement of LEA_4 structural and functional properties is likely encoded in specific patterns of their physicochemical characteristics.

## 1. Introduction

For a long time, a protein’s function has been associated with its uniquely folded structure. By contrast, intrinsically disordered proteins (IDPs) lack a defined three-dimensional (3D) structure, yet appear to be prevalent and functional. The incidence of IDPs has been recognized since the 1990s [1,2] and their high abundance in all life kingdoms has become more and more apparent over the last two decades. Over 40% of all eukaryotic proteins are predicted to be intrinsically disordered or to contain long disordered regions [3]. They function in transcriptional regulation, translation, cellular signal transduction [4] and molecular chaperoning [5]. The structure-function relationship of IDPs is complex and spans a broad spectrum of possibilities. While some IDPs are fully functional in the disordered state, acting as entropic chains, others undergo major conformational changes upon partner binding [6]. Protein intrinsic disorder is also abundant in plants [7], with functions in signaling networks, the control of metabolic processes, transcriptional regulation and the response to biotic and abiotic stresses (see [8,9] for recent reviews).

In the context of abiotic stress responses, late embryogenesis abundant (LEA) proteins are of special importance. They are expressed in plants, bacteria and several species of invertebrate animals mostly under conditions leading to cellular dehydration [10]. Their presence has been widely associated with cellular tolerance to dehydration, induced for example by freezing, high salinity or drying [11]. Although they are typical IDPs in dilute solution, several LEA proteins (partially) fold into mainly α-helical structure in response to desiccation or partial dehydration [12,13,14]. While the available literature agrees on a protective function of LEA proteins during desiccation and dehydration at a physiological level [15], their molecular functions are still largely unknown. Various molecular functions in plant abiotic stress tolerance have been suggested for LEA proteins, all related to counterbalancing damage resulting from cellular dehydration [16], for example the stabilization of cytoplasmic sugar glasses [17], the stabilization of enzymes against stress-induced aggregation and inactivation (see [16,18] for reviews), the stabilization of membranes [19,20] and the sequestration of divalent ions [21]. The vast majority of experimental evidence for these functions stems from *in vitro* experiments. Several LEA proteins are able to provide improved tolerance to drying or freezing when recombinantly expressed in bacteria or plants [22]. However, information on their actual molecular function *in vivo* is limited and studies are complicated by the fact that LEA protein function likely directly depends on the hydration level of the system. 

In the model plant *Arabidopsis thaliana*, 51 LEA protein encoding genes were identified and grouped into nine families based on Pfam domains [23]. The LEA_4 family is the largest and shows the highest sequence diversity. Based on sequence information, the *A. thaliana* LEA_4 family (also classified as group3/group5, D7/D29) was previously characterized in terms of molecular mass, hydropathy and sequence similarity [23]. Experimentally, LEA_4 proteins have been localized in all cellular compartments [24]. The LEA_4 family is also the largest and most heterogeneous LEA Pfam family in other plants species, such as maize [25] and poplar [26]. In addition, the majority of LEA proteins from non-plant organisms belongs to the LEA_4 group [18]. LEA_4 proteins are particularly abundant in seeds (12 of the 18 LEA_4 genes in *A. thaliana* are expressed in seeds) [23], which has led to postulating a functional role in seed desiccation tolerance [25,27]. 

A conserved 11 amino acid motif (TAQAAKEKAXE) was initially suggested as a signature for LEA_4 family proteins in cotton [28], but is underrepresented in the *A. thaliana* LEA_4 family [23]. Next to the overrepresentation of charged amino acids and underrepresentation of cysteine, phenylalanine, isoleucine, leucine and tryptophan, which holds for LEA proteins in general, the LEA_4 group is characterized by a high abundance of alanine and a low abundance of glycine compared to other LEA families, seemingly resulting in a high predicted α-helical secondary structure content, compared to other LEA Pfam families [29]. Indeed, structural studies of several LEA_4 proteins indicate that they are fully unstructured in dilute solutions, but undergo structural transitions towards mainly α-helical structure in the presence of osmolytes such as sucrose, glycerol and ethylene glycol (EG), in organic solvents such as 2,2,2-trifluoroethanol (TFE) or methanol, and during dehydration. However, a similar behavior has been reported for LEA proteins from other families [12,13,14]. Whether the tendency of α-helix formation is higher in the LEA_4 family as a consequence of the high alanine and low glycine content awaits future studies. 

We have contributed several studies on the LEA_4 proteins COR15A, COR15B, LEA25 and LEA11, all stemming from the same homology clade [19,30,31,32,33]. All are essentially IDPs in the fully hydrated state, show a coil-helix transition in response to desiccation and partial dehydration and stabilize liposomes modeling the lipid composition of inner chloroplast membranes during a freeze/thaw cycle. We have provided evidence that COR15A and COR15B protect chloroplast and plasma membranes during freezing *in vivo*, but do not stabilize chloroplast enzymes under these conditions [19]. The association of COR15A with membranes requires the transition from a random coil to an at least partially α-helical structure [19,32,34]. LEA7, another *A. thaliana* LEA_4 protein from a different clade, on the other hand, stabilizes the enzyme lactate dehydrogenase as well as enzymes in the *A. thaliana* soluble leaf proteome during freezing and drying [35]. Expression of LEA7 in *Escherichia coli* improves survival under cold, but non-freezing conditions and in response to osmotic stress [36], while it enhances survival of yeast cells (*Saccharomyces cerevisiae*) specifically during drying [37].

A major drawback in the study of LEA protein function and structure-function relationships is that most functional data originate from the study of single LEA proteins rather than from systematic, comparative analyses. Here, we therefore widened our previous approach and undertook a systematic bioinformatics study on all 18 members of the *A. thaliana* LEA_4 family. In addition, we experimentally characterized six members of two distinct, lowly homologous clades of LEA_4 family proteins, including those described above. Finally, we compared their structural and functional characteristics to shed new light on how structure and function may be encoded in LEA protein sequences.

## 2. Results

### 2.1. Bioinformatic Characterization of the A. thaliana LEA_4 Family 

**Figure 1:**
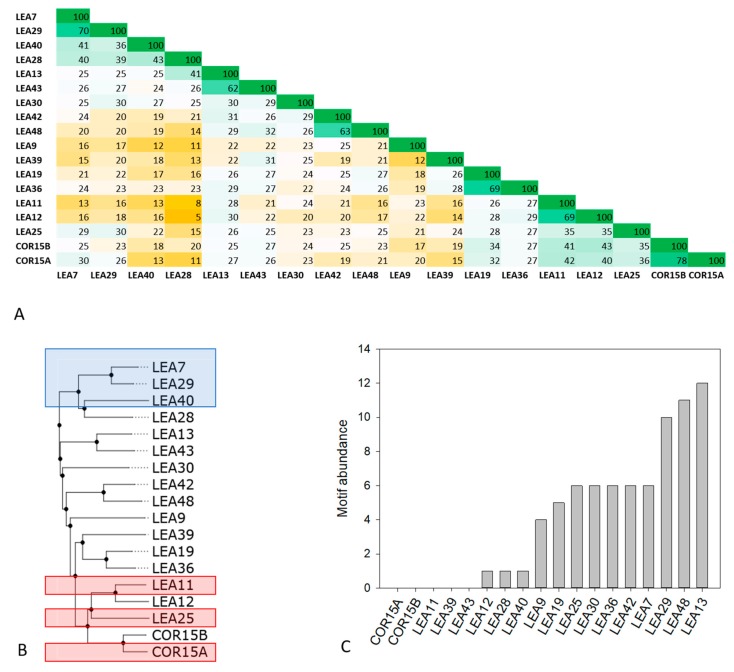
Percent identity matrix (**A**) and dendrogram (**B**) of all 18 members of the late embryogenesis abundant (LEA)_4 Pfam family from *Arabidopsis thaliana*. In A, green colors indicate high, yellow colors low sequence identity. Proteins chosen for experimental analysis are boxed in blue (LEA7 clade) and red (COR15A clade) in B. Abundance of the conserved 11-mer motif TAQAAKEKAXE in the 18 LEA_4 proteins calculated with the FIMO tool is shown in (**C**).

The LEA_4 Pfam family in Arabidopsis covers a wide range of molecular masses (from 7 to 67 kDa) and representatives of the family are localized in all subcellular compartments (Table S1). Sequence identities within the LEA_4 Pfam family were calculated using multiple sequence alignment followed by percent identity scoring (Figure 1A). 

With an average sequence identity score of 25 ± 4%, the LEA_4 family does in general not display high sequence conservation. Exceptions are the protein pairs LEA11/12 (69% identity) and COR15A/COR15B (78% identity), which are encoded by tandem repeat gene pairs resulting from local duplication events [23]. Beyond these, sequence identity above 45% is only present pairwise in LEA42/48, LEA19/36, LEA13/43 and LEA7/29, which are encoded by homeologous gene pairs resulting from whole genome duplication events [23].

The abundance of one or multiple repeats of the 11-mer motif TAQAAKEKAXE has been suggested to be highly conserved in the LEA_4 family from cotton [28]. Figure 1C shows the abundance of this motif in the *A. thaliana* LEA_4 family. Apparently, motif conservation is much lower in LEA_4 proteins from *A. thaliana* than from cotton [23]. Five out of the 18 proteins do not possess the motif at all. Three more proteins have one single copy of the motif and only half of the proteins feature five or more motif repeats. The highest abundance of the motif relative to sequence length is found in LEA29 with 10 repeats, constituting 50% of the complete protein sequence. As this motif has been predicted to be involved in coil-coil formation [28,29,38], we used the Deepcoil prediction server to screen the LEA_4 protein sequences for the presence of putative coiled coil domains (Figure S1). We find a reasonable agreement of sequence segments presenting putative coiled coil domains and the 11-mer motif. However, in several cases coiled coil was also predicted for sequence domains that do not constitute the 11-mer motif (Figure S1).

**Figure 2:**
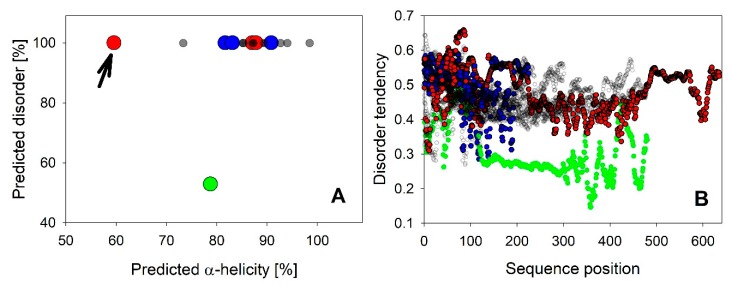
Overall disorder tendency predicted by CSpritz was plotted against α-helicity predicted by Porter 5.0 for all 18 *A. thaliana* LEA_4 proteins in **A**. LEA25 is indicated with an arrow. **B** shows disorder tendency per residue plots of all proteins. LEA9 is indicated in green in A and B. LEA7 clade members are plotted in blue, COR15A clade members in red in A and B.

It is not surprising that the majority of all LEA_4 proteins is predicted to be fully disordered (Figure 2A). However, it is interesting to note that the disorder probability of each residue is only around 50% throughout the complete sequence (Figure 2B). Therefore, a universal feature of these proteins may be that they are just on the brink of disorder, as previously suggested for the LEA_4 protein COR15A [39]. This applies for all LEA_4 proteins except LEA9, which has an overall predicted disorder of only about 50% and a residue disorder tendency between about 20% and 40%. Using secondary structure prediction, we found that all LEA4 proteins are predicted to be α-helical, with in most cases 80% to 100% α-helicity (Figure 2A). LEA25, which is by far the LEA_4 protein with the highest molecular mass (Table S1), is an exception with only 60% predicted helicity (Figure 2A, arrow). A similar prediction pattern, combining high disorder probability with a high propensity for α-helix formation, has been reported for LEA proteins previously by Janis et al. [40], who suggested that such a prediction pattern might indicate folding potential.

We have previously shown that COR15A folds into amphipathic α-helices in response to reduced water availability [14,31], which drives membrane association and stabilization [32,41]. This structural element requires a regular distribution of hydrophobic amino acids along the protein sequence, usually every N+3 and/or N+4 positions, separated by polar residues [42]. We used the CIDER tool to calculate linear hydropathy plots for all 18 *A. thaliana* LEA_4 proteins (Figure S2). All sequences show a regular distribution of hydropathy with a periodicity similar to that of COR15A, indicating a similarly amphipathic character of the putative α-helices of the remaining 17 proteins.

**Figure 3:**
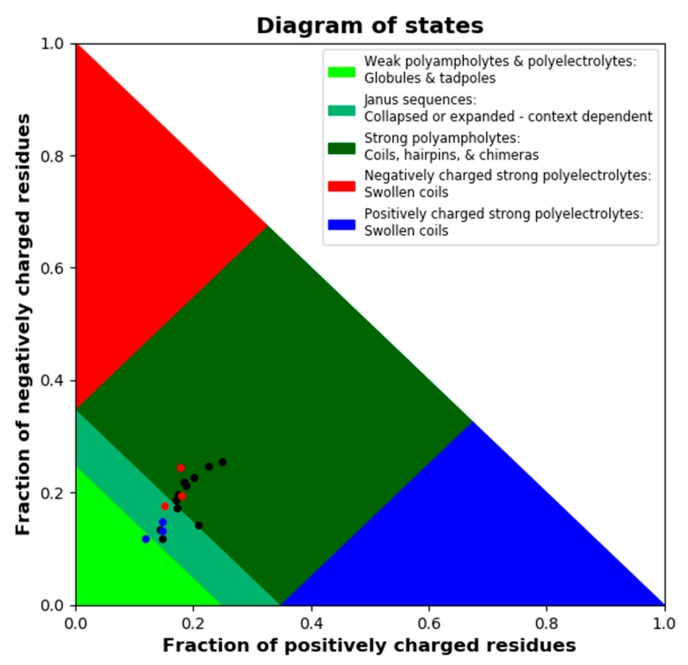
Das-Pappu phase diagram calculated using local CIDER including all 18 *A. thaliana* LEA_4 proteins. LEA7 clade members are plotted in blue, COR15A clade members in red, the remaining LEA_4 proteins in black.

LEA proteins are further characterized by a high fraction of charged residues. We used the CIDER tool to dissect charge distribution and segregation along the sequences of all 18 LEA_4 proteins (Figure S3). All possess a high number of charged amino acids, but carry a net charge close to zero (Table S2). The parameter κ defines charge distribution symmetry with a value of 1 reporting perfect segregation of positive and negative charges along the primary sequence and a value of 0 stating a perfect mixing of positive and negative charges [43]. All 18 LEA_4 proteins show an almost perfectly even distribution of positively and negatively charged amino acids defined by a κ value close to zero (Table S2), indicating the underrepresentation of long-range interactions between oppositely charged blocks [43]. Figure 3 depicts a Das-Pappu phase diagram, which suggests structure-ensemble relationships based on the incidence of the fractions of negatively and positively charged residues. 

The 18 LEA_4 sequences show a narrow distribution with a classification as Janus sequences or strong polyampholytes. It is interesting to note that the fractions of positively and negatively charged residues are almost perfectly well balanced in all LEA_4 proteins, resulting in a net charge per residue of around zero. However, both fractions are slightly smaller in the LEA7 clade compared to the COR15A clade. 

### 2.2. Experimental Structural Characterization of the Members of two LEA_4 Clades

As *in silico* sequence analysis indicated highly similar physicochemical characteristics of the LEA_4 proteins, we decided to take a closer look at structural and functional properties of the two most distant clades within the LEA_4 Pfam family (Figure 1B), comprising three proteins each. In the following, these will be called the LEA7 clade (LEA7, LEA29 and LEA40) and the COR15A clade (COR15A, LEA11, LEA25). It should be mentioned that the COR15A clade contains two more proteins, LEA12 and COR15B. We did not include these two proteins in our analysis, because they are highly similar to LEA11 and COR15A, respectively (Figure 1A,B). 

The six experimentally characterized LEA proteins not only represent LEA_4 proteins from phylogenetically distant clades of this family (Figure 1A), but also show a wide range of expression patterns and subcellular localizations (Table S1). The gene encoding the chloroplast stromal protein COR15A is expressed in various non-seed tissues mainly in response to low temperature. The gene encoding the second chloroplast protein, LEA11, is exclusively expressed in flower buds and is not responsive to low temperature [23]. Expression of the gene encoding the nuclear and cytosolic LEA40 is limited to reproductive tissues. Expression of the genes encoding the other two nuclear and cytosolic proteins (LEA29 and LEA7) are either expressed exclusively in seeds, or in buds, seeds and generally in response to stress. Finally, LEA25 is localized in the cytoplasm and the corresponding gene is expressed in seeds and in vegetative tissues in response to high salt concentrations. We have previously described structural and functional details of four of the six proteins (COR15A, LEA7, LEA11, LEA25) [14,19,30,31,33,34,35,39,44], but a comprehensive comparative analysis has not been provided.

Simultaneous static and dynamic light scattering (SLS/DLS) measurements were conducted to determine the apparent average single particle mass (M_app_) and the apparent average hydrodynamic radius (R_S_) of the proteins in solution as a function of concentration (Figure S4, [30]). Absolute molecular mass and R_S_ were obtained by extrapolation of apparent molecular mass and R_S_ to infinite dilution. All investigated LEA proteins are essentially monomeric under fully hydrated conditions. Compared to globular proteins, R_S_ of all investigated LEA proteins was rather large, indicating their expanded, non-compact nature.

**Figure 4:**
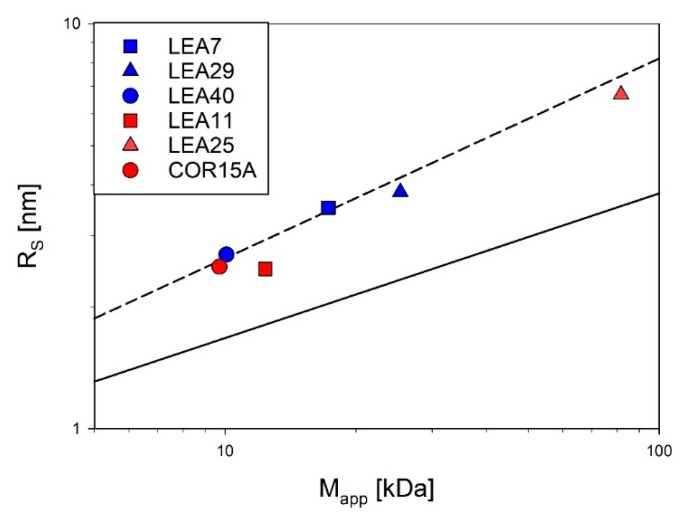
Compactness of the six LEA_4 family proteins visualized by using scaling laws of the type R_S_ = a * M^b^. The Stokes radius (R_S_) was determined by dynamic light scattering (DLS) and plotted against the molecular mass determined by static light scattering (SLS). The average compactness according to the scaling laws for compactly folded globular proteins (solid line [45]) and intrinsically disordered proteins (dashed line [46]) are shown for comparison. Data for COR15A, LEA11 and LEA25 were taken from a previous publication [30].

Accordingly, Figure 4 shows R_S_ as a function of the molecular mass for the six investigated LEA proteins in log-log representation in relation to the scaling behavior of globular proteins and IDPs under fully hydrated conditions. All essentially follow the scaling law for IDPs with the exception of LEA11, which seems slightly more compact.

**Figure 5:**
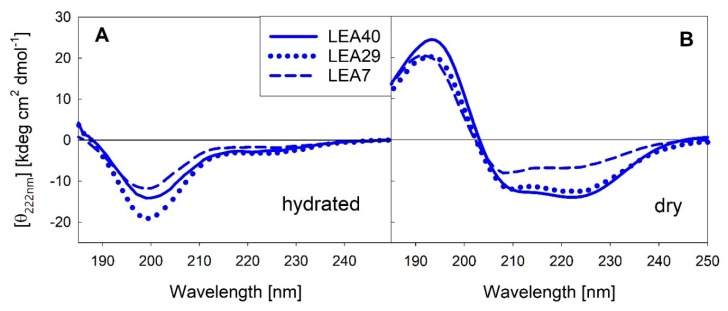
Far-UV circular dichroism (CD) spectra of the three LEA_7 clade proteins in the fully hydrated state (**A**) and after drying (**B**). All spectra were averaged over at least three replicates per sample.

Various reports from our and other groups indicate that several LEA proteins are unstructured in solution, but adopt mainly α-helical structure upon full or partial dehydration. We measured far-UV circular dichroism (CD) spectra of the three proteins contained in the LEA7 clade in the fully hydrated state and after complete desiccation (Figure 5). The corresponding spectra for the three proteins of the COR15A clade have been published previously [30].

As already reported for the COR15A clade, the LEA7 clade proteins are essentially disordered in dilute solution and fold into mainly α-helical structure upon desiccation. α-helical structure is less distinct in LEA7 compared to the other proteins. 

**Figure 6:**
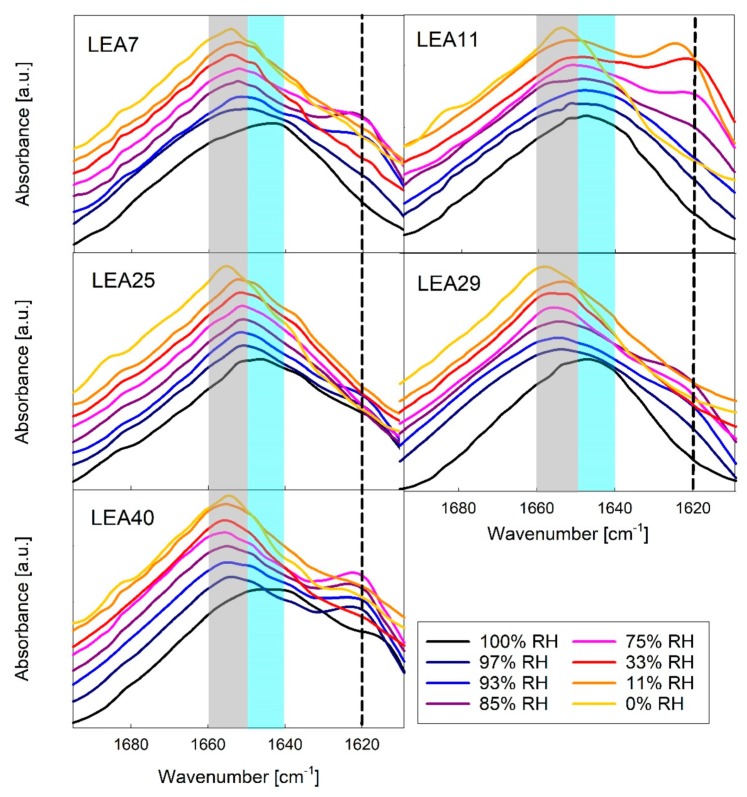
Amide I region of Fourier-transform infrared (FTIR) spectra of five out of the six LEA_4 family proteins in different relative humidity (RH) conditions. Peak maxima indicate unstructured proteins (1650–1640 cm^−1^, shaded blue), α-helix (1660–1650 cm^−1^, shaded grey) and β-sheet aggregates (about 1620 cm^−1^, dashed line). The spectra in each panel are offset from each other for better visibility. A corresponding dataset for COR15A has been published previously [34].

*In vivo*, not all LEA proteins are subjected to full desiccation, but rather to partial dehydration, for example under drought or freezing conditions. We therefore monitored secondary structure of the six LEA_4 proteins in response to decreasing relative humidity (RH) using the Amide I band of Fourier-transform infrared (FTIR) spectra (Figure 6). This absorbance band mainly results from the C=O stretching vibration and is directly related to the protein backbone conformation.

A maximum at 1650–1640 cm^−1^ indicated that all proteins were mainly disordered at 100% RH, which is in line with the CD data. Structural transitions of LEA_4 proteins were resolved at different RH conditions with a coil-helix transition indicated by a shift of the Amide I peak maximum to higher wavenumbers. At intermediate RH, a shoulder at 1620 cm^−1^ indicated the formation of minor amounts of intermolecular β-sheet aggregates in all LEA_4 proteins. Interestingly, these aggregates were not present at 0% RH and were dissolved upon further increasing RH.

Coil-helix transitions in LEA proteins have been reported not only in response to drying, but also with increasing solution osmolarity using for example high concentrations of glycerol and EG. Figure S5 shows FTIR spectra of the six LEA_4 proteins in EG concentrations ranging from 0 to 12 osM. Similar to decreasing RH, increasing osmolarity induces coil-helix transitions, indicated by a shift of the Amide I maxima to higher wavenumbers. Aggregation in terms of intermolecular β-sheet formation was less prominent than at intermediate RH. Nevertheless, very high solution osmolarities above 9 osM induced some aggregation in LEA11 and LEA25, but not in the other LEA proteins.

**Figure 7:**
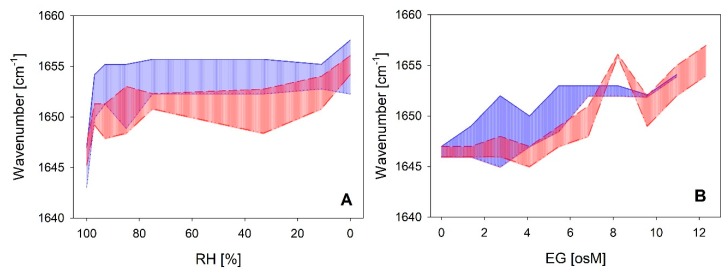
Variance in the FTIR Amide I peak maxima of the two LEA_4 clades as a function of RH (**A**) and ethylene glycol (EG) (**B**), respectively. Highest and lowest measured wavenumber in the peak maxima within the LEA7 clade (blue) and the COR15A clade (red) represent the limits of the colored areas.

A direct comparison of the coil-helix transitions between both LEA_4 clades as a function of RH and EG is shown in Figure 7. The wavenumber shift was most prominent already at rather high RH with an equilibrium reached at 93–85%. The transition was similar in both clades in response to decreasing RH (Figure 7A). However, at intermediate and low RH, the Amide I peak maxima of the proteins of the LEA7 clade were on average around 5 cm^−1^ higher than those of the proteins of the COR15A clade, indicating a shift in the coil-helix equilibrium towards a higher ratio of α-helical structure in the former case. The corresponding transitions in increasing concentrations of EG (Figure 7B) are characterized by similar minimum and maximum peak wavenumbers at the lowest and highest EG concentrations between both clades. However, higher EG concentrations are necessary to induce the coil-helix transition in the proteins of the COR15A clade compared to the LEA7 clade, which is indicated by a shift of the respective area along the x-axis.

**Figure 8:**
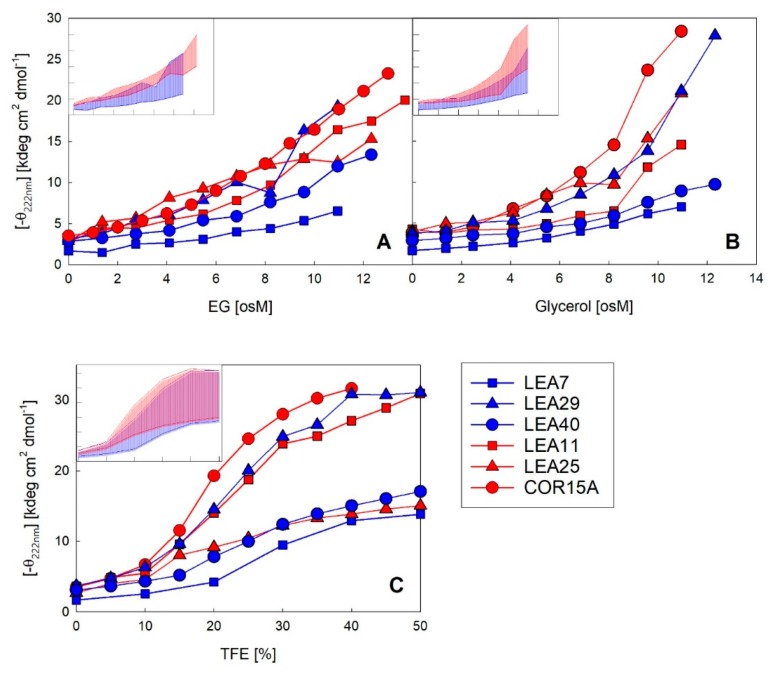
Increase in α-helicity plotted as negative ellipticity at 222 nm of the six LEA_4 family proteins as a function of EG (**A**), glycerol (**B**) and TFE (**C**) concentration. The datasets for COR15A in the presence of all three solutes and for LEA11 and LEA25 in glycerol and TFE have been published previously [30,33]. Insets show the variance in negative ellipticity at 222 nm of the two LEA_4 clades. Highest and lowest ellipticity at 222 nm within the LEA_7 clade (blue) and COR15A clade (red) represent the limits of the colored areas. Axis labels and scales are identical to the corresponding full-size panels.

As a complementary method to FTIR, we used far-UV CD spectroscopy to monitor coil-helix transitions of the six LEA_4 proteins in increasing solution osmolarity using the osmolytes glycerol and EG. In addition, we used increasing concentrations of TFE, which triggers the maximum propensity of a given protein to form α-helical structure (Figure 8). All proteins showed typical random coil spectra in dilute solution. With increasing concentrations of each of the co-solvents, the changes in the spectral shape report on the formation of ordered secondary structure. In EG (Figure S6), four out of the six proteins showed a two-state transition, indicated by the isodichroic points and the typical α-helical spectral shape with two negative maxima at 208 and 222 nm at high EG concentrations. This did not apply to LEA25, which displayed a spectral shape indicative of the presence of other secondary structure elements besides α-helix and the absence of an isodichroic point. This was also true in glycerol solutions, as published previously [30]. LEA7 showed some spectral changes in response to increasing EG concentrations. However, these changes were rather small, with an α-helix content just below 15% at an EG concentration of 11 osM, compared to the other LEA proteins, which reached an average α-helicity of 45% under these conditions. 

The coil-helix transitions were similar in EG (Figure 8A) and glycerol (Figure 8B) for all six LEA_4 proteins, except at very high osmolarities (>8 osM) in the case of COR15A and LEA25, where α-helicity strongly increased in glycerol, but not in EG. A coil-helix transition was similarly apparent in increasing concentrations of TFE (Figure 8C). We observed a clear separation of two subsets of LEA proteins in TFE, one including COR15A, LEA11 and LEA29, which at high TFE concentrations reached an average α-helicity of almost 100%. The other subset, comprising LEA7, LEA25 and LEA40, showed an average α-helicity of only about 40–50% in 50% TFE. Interestingly, the strong folders had a higher folding potential than they realized in response to high solution osmolarity, as evident when comparing folding in TFE and EG. By contrast, the weak folders seemed to fully exploit their folding capacity in response to high solution osmolarity, again comparing TFE and EG. The distinction between strong and weak folders in TFE did not correspond to the two clades of LEA_4 proteins. Each clade included at least one strong and one weak folder. Accordingly, the variance within the clades (Figure 8, insets) was high and both clades widely overlapped with a small tendency towards higher α-helicity for the COR15A clade. 

### 2.3. Structural Characterization of the two LEA_4 Clades by MD Simulation

**Figure 9:**
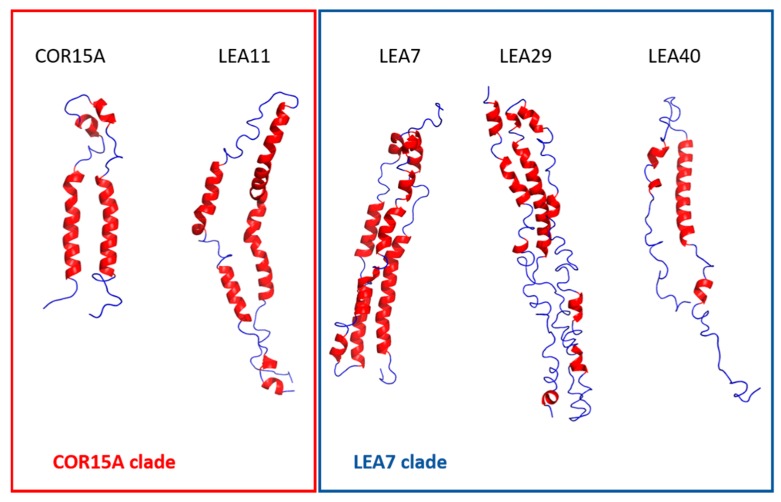
Representative models after 500 ns molecular dynamics (MD) simulations in 100% glycerol in ribbon style. α-helices are colored in red, random coil in blue.

**Figure 10:**
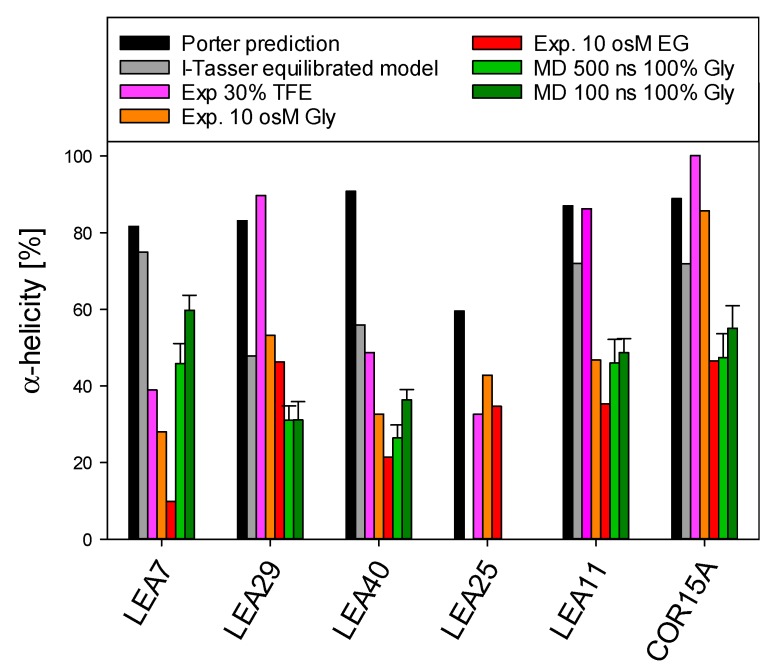
Comparison of α-helical structure of the six LEA_4 proteins from prediction, MD simulation and experiment. Error bars depict SD of five simulation replicate trajectories. Gly—glycerol.

We predicted 3D models of five LEA_4 proteins using I-TASSER. To obtain a representation of the natively folded state of the chosen proteins, we used 500 ns molecular dynamics (MD) simulations to equilibrate these models in 100% glycerol (Figure 9), comparable to our earlier reports on COR15A [31]. The simulation approach was suitable for five out of the six proteins due to their relatively small number of amino acids, but was not realizable for the 635 amino acid protein sequence of LEA25. All structural models are characterized by α-helical segments separated by disordered sequence stretches. For COR15A, such a model was experimentally validated by NMR spectroscopy in 20% TFE, indicating two helical segments connected by a disordered loop [39]. Figure 10 shows a comparison of the fraction of α-helical structure of the six LEA_4 proteins from prediction, MD simulation and experiment. 3D modeling and MD information is not available for LEA25 due to its large molecular mass. Both secondary structure prediction and I-TASSER models largely overestimated the amount of α-helicity of all proteins at high solution osmolarity. In the presence of 30% TFE, LEA29, LEA11 and COR15A approached α-helicities close to the predicted values. This was not true for LEA7, LEA40 and LEA25, where α-helicity was relatively low in 30% TFE. In the MD simulations, the equilibration of the 3D models in 100% glycerol corresponded closely to the experimentally determined α-helicity at high solution osmolarity. Exceptions were LEA7 and LEA29, the largest proteins used in our modeling approach, and COR15A, where the α-helicity of the models after MD simulation closely matched the experimental data in EG, but underestimated helicity in glycerol. Starting from the structure *in vacuo*, all models partially unfolded in the first 30–50 ns after transfer to 100% glycerol. After this initial fast change, the root mean square deviation (RMSD) slowly increased further over the remaining simulation time, indicating that the simulation parameters were not sufficient to reach a stable equilibrium (Figure S7). We calculated the average helicity over trajectories from five simulation replicates after 100 ns and 500 ns to estimate if the unfolding proceeded during this second slow progression of the RMSD. Apparently, it was minor for LEA11 and LEA29 and around 10% for COR15A, LEA40 and LEA7. 

### 2.4. Temperature Effects on Folding and Cryoprotective Activity of the LEA_4 Proteins

**Figure 11:**
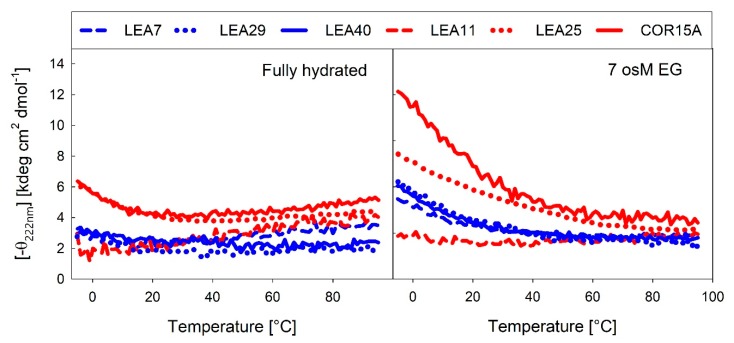
Temperature-dependent changes in α-helicity of the LEA_4 proteins, expressed as the negative ellipticity at 222 nm under fully hydrated conditions (**A**) and in 7 osM EG (**B**).

Several LEA proteins accumulate in response to low temperature and may stabilize cellular components during freezing. COR15A is a well-described example of how membrane stabilization increases plant freezing tolerance *in vivo* [19]. Therefore, we recorded temperature dependent changes in α-helicity using the far-UV CD signal at 222 nm under fully hydrated conditions (Figure 11A) and at high solution osmolarity in 7 osM EG (Figure 11B).

In the fully hydrated state, a temperature increase above room temperature (RT) induced a small degree of α-helical structure, which has been reported for IDPs previously [47]. This was apparent in COR15A, LEA11 and LEA7. As expected, at 7 osM EG, a temperature increase above RT resulted in unfolding of the α-helical structure, which was completed at about 60 °C. In the fully hydrated state, LEA25, COR15A and to a lesser extent also LEA29 showed an inflection point around 20 °C. Lowering the temperature below RT triggered formation of α-helical structure, indicating that COR15A, LEA25 and LEA29 structurally reacted to temperature changes in a physiological range from ambient down to subzero temperatures, which was not evident for LEA7, LEA11 and LEA40. This was even more pronounced at high solution osmolarity. All proteins except LEA11 showed an increase in α-helicity with decreasing temperature at 7 osM EG. The inflection point was additionally shifted up to 50-60 °C. The three proteins from the LEA_7 clade showed an almost identical temperature dependent moderate increase of α-helicity at 7 osM EG. By contrast, there were pronounced differences among the proteins of the COR15A clade, with COR15A showing a strong gain of α-helical structure up to 60% at −5 °C, while LEA11 did not show any structural reaction to temperature at all.

**Figure 12:**
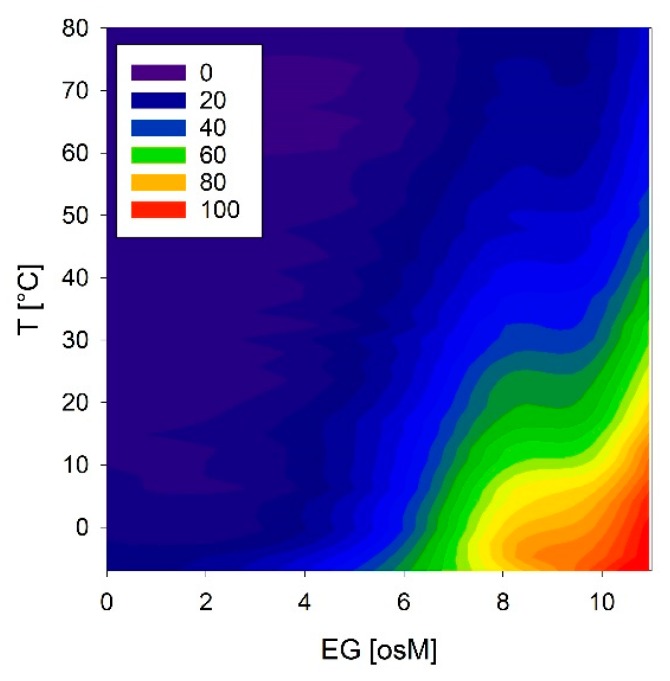
Temperature and osmolarity dependent conformational changes of COR15A estimated from the ellipticity at 222 nm. Color codes correspond to the percentage of α-helicity.

We investigated these conformational reactions to both temperature and osmolarity in more detail using COR15A, which showed the strongest effects. Temperature scans were obtained at solution osmolarities ranging from 0 to 11 osM EG (Figure 12). COR15A was able to access the full conformational space ranging from complete random coil to 100% α-helicity in response to the modulation of temperature and osmolarity. It is interesting to note that the combinations of both parameters resulting in the highest α-helicities were apparently close to the environmental situation the protein might experience *in vivo* during mild freezing.

**Figure 13:**
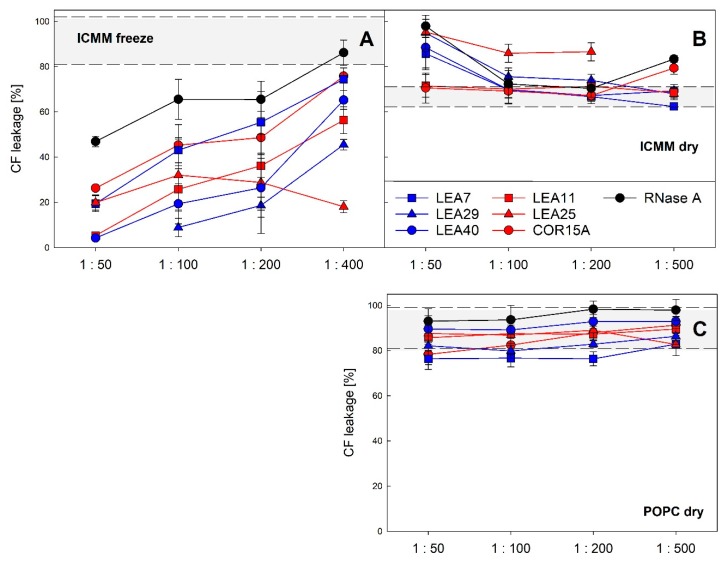
Liposome stability after a freeze/thaw cycle (**A**) or a dehydration/rehydration cycle (**B**,**C**). The integrity of large unilamellar ICMM vesicles, composed of 40% monogalactosyldiacylglycerol (MGDG), 30% digalactosyldiacylglycerol (DGDG), 15% sulfoquinovosyldiacylglycerol (SQDG) and 15% egg phosphatidylglycerol (EPG) (**A**,**B**) or of pure 1-palmitoyl-2-oleoyl-phosphatidylcholine (POPC) (**C**) was assayed as leakage of the soluble marker CF. The samples contained the indicated LEA_4 proteins or RNase A as reference protein at different protein-lipid mass ratios. Grey shaded areas depict average values for liposomes frozen or dried in the absence of protein, limited by ± SD. The averages of three replicates from one to three independent experiments are shown ± SD.

We have previously reported that LEA proteins act as membrane stabilizers during a freeze/thaw cycle *in vitro* [19,30] and that in the case of COR15A this translates to a function in increasing freezing tolerance *in vivo* [19]. Therefore, we analyzed the stability of liposomes modeling the lipid composition of inner chloroplast membranes (ICMM) after a freeze/thaw cycle and a dehydration/rehydration cycle in the presence of all six LEA_4 proteins at different LEA protein: lipid mass ratios (Figure 13). In the latter case we used liposomes made of pure POPC (1-palmitoyl-2-oleoyl-phosphatidylcholine) as a specificity control.

As reported previously, all three proteins constituting the COR15A clade stabilized ICMM liposomes during a freeze/thaw cycle [30]. The same was true for the three proteins of the LEA7 clade. In most cases, the degree of stabilization depended on the protein: lipid ratio, suggesting that vesicle stabilization is related to vesicle surface coverage. The most efficient stabilizers were LEA29, LEA11 and LEA40, which showed a similar performance, followed by COR15A and LEA7. LEA25 performed differently, as it stabilized liposomes independent of protein concentration in the investigated range of protein:lipid ratios. The differences in vesicle protection observed among the six proteins were not related to membership in the two clades. During dehydration/rehydration none of the six LEA_4 proteins showed a stabilizing effect for either ICMM or POPC liposomes. In contrast to freezing, LEA25, LEA29 and LEA40 even destabilized ICMM liposomes at high protein concentrations under these conditions. While LEA proteins affected ICMM stability under both treatments, they had no significant effect on POPC stability during a dehydration/rehydration cycle.

## 3. Discussion

LEA proteins are widely considered as protectants during abiotic stresses involving cellular dehydration. Their disordered nature at full hydration and folding in response to various experimental conditions have been probed in numerous studies. In addition, several molecular functions have been suggested, mainly the stabilization of membranes and labile enzymes during drying and freezing. It is, however, largely unclear, whether folding is required for functionality and how physicochemical characteristics of the proteins modulate their structure and function. These questions are best addressed by systematic analyses covering a sufficiently large number of LEA proteins spanning a wide range of sequence diversity. We focused on the LEA_4 Pfam family from *A. thaliana*, which lacks high sequence similarity among its members and investigated three members each of two distinct, low-similarity LEA_4 family clades. We present structural analyses and a functional investigation of liposome stabilization during freeze/thaw and dehydration–rehydration cycles. These experimental data were combined with information on physicochemical characteristics of all 18 LEA_4 family members obtained through a bioinformatic approach to shed light on potential determinants of LEA_4 protein folding and functionality. 

All six investigated LEA_4 proteins are IDPs in the fully hydrated state that show structural transitions in response to dehydration, low temperature, increasing solution osmolarity and the structure inducing alcohol TFE. Folding in response to dehydration, osmolarity and TFE has been previously reported for many LEA proteins from different Pfam families. However, folding into α-helical structure in response to low temperature has not been shown for LEA proteins before. In the fully hydrated state, this effect was small and only obvious for COR15A, LEA25 and LEA29. It was strongly amplified at high osmolarity. Folding at low temperatures *per se* is not surprising, as transiently formed α-helices in IDPs are stabilized under these conditions [48], probably due to the decreasing entropy. Low-temperature induced folding has, for example, been described for type I fish antifreeze proteins [49,50] and small α-helical peptides [51]. 

In response to dehydration, five of the six LEA proteins showed a transition to mainly α-helical structure, indicated by the shift in the Amide I peak position to higher wavenumbers and the shape of the CD spectra with two negative maxima at 222 and 208 nm. LEA25 was an exception, with a single broad negative maximum at 218 nm in the CD spectra and a shoulder at 1620 cm^−1^ in the Amide I peak at high osmolarity indicating the presence of additional secondary structure elements and/or β-sheet aggregates. For the other LEA proteins, a two-state folding process was indicated by the isodichroic points at 205 nm in the CD spectra. The coil-helix transition was cooperative in response to decreasing RH. In contrast, decreasing temperature and increasing solution osmolarity resulted in an uncooperative transition in all six proteins. Increasing concentrations of TFE induced a coil-helix transition with apparent intermediate cooperativity. These differences can be explained considering the different folding mechanisms triggered by the experimental conditions. Lowering the water activity is the effect common to lowering RH and increasing cosolute concentrations [52]. However, in addition to their impact on water activity, osmolytes are preferentially excluded from the proteins. This is the consequence of mutual perturbations of the chemical potentials of protein and cosolute [52]. Contact of the cosolute with a protein is unfavorable in the folded and in the unfolded state, but more unfavorable for the latter (with a transfer free energy higher in the unfolded than in the folded state), thus resulting in stabilization of protein structure [53]. Although the mechanism by which α-helical structure is stabilized by TFE is still not completely understood, it is clearly different from the osmolytes. TFE induces preferential solvation of proteins, thus stabilizing intramolecular H-bonds [54]. This is maximal around 30% TFE [55], which may explain the levelling off in the transition curves, indicating apparent cooperativity in the folding of all six proteins above 30% TFE. 

Well-folded globular proteins usually show highly cooperative folding and unfolding transitions, in the simplest case following a two-state process, where only the unfolded and the folded states are populated. Such a two-state folding/unfolding process is characterized by a relatively high energy barrier. This allows globular proteins to tolerate physiologic variations in temperature and solvent environment [56]. The other extreme of possible folding/unfolding scenarios is a highly uncooperative gradual barrier-less “downhill” transition, characterized by a minimal amount of stabilizing intramolecular interactions, with the ensemble of structures becoming progressively folded [57,58]. IDPs have been defined as “one-state gradual downhill folders”. In this context, the term molecular rheostat has been coined to describe their gradual conformational changes [57]. With their gradual, non-cooperative conformational reaction to temperature and solution osmolarity, the six LEA_4 proteins exhibit downhill-folder characteristics. Moosa et al. discussed that some IDPs, due to unfavorable energetics, might be unable to adopt compact folded structures under physiological conditions, making the denatured state the predominantly populated conformational ensemble [59]. In this context, LEA proteins could be considered as exceptionally unstable folded proteins under standard physiological conditions, maybe due to the underrepresentation of hydrophobic interactions. Since LEA proteins mostly function under conditions of cellular dehydration, their “native” state might well be considered as the high-osmolarity α-helical conformation. In addition, the conformational space of five out of the six LEA proteins was not only modulated by osmolarity, but also by low temperature. This was most pronounced for COR15A, which functions as a natural cryoprotectant. Such conformational adjustment is potentially not limited to osmolarity and temperature. Conformational changes of COR15A, LEA11 and LEA25 are additionally modulated by the presence of membranes at high solution osmolarity [19,30]. Further factors may be envisioned that lead to a multidimensional conformational fine-tuning. In such a scenario, folding could be triggered rapidly in response to cellular needs modulated by changing environmental conditions. 

Folding in increasing concentrations of EG was monitored by FTIR and CD spectroscopy. While the FTIR data indicated a structural transition with recognizable pre- and post-transition plateaus, the CD data pointed towards uncooperative folding without post transition baselines. Moreover, transition midpoints of the two LEA clades in FTIR differed, with the transitions of LEA7 clade proteins occurring at lower EG concentrations than those of the COR15A clade. Transition midpoints were not present in the CD data, but folding was more pronounced at any given EG concentration for the COR15A clade. This discrepancy is likely due to the fact that we used the position of the Amide I peak maximum as a proxy for the coil-helix transition. However, this peak is composed of several bands indicative of different secondary structure components. The overall peak position is therefore not sufficient for absolute quantification of α-helix content, but rather indicates relative shifts in the folding state. The apparent differences between the two clades in the FTIR data can most likely be explained by differences in the peak width (Figure S8), which is a function of the distribution of the underlying component peaks. Additionally, contributions from side chain absorption can occur, which may impact spectral characteristics of the Amide I band [60]. Specifically, this concerns the charged residues glutamic and aspartic acid and lysine, which are overrepresented in the COR15A clade and glutamine, which is more abundant in the LEA7 clade [61]. This may also have contributed to the difference in Amide I peak positions between the two clades in the RH measurements. CD spectroscopy is better able to quantify the α-helical content and the coil-helix transition, while IR is clearly able to detect the secondary structure transition in a given protein, but the differences among the proteins should be treated with caution. 

Conformational changes induced by TFE, osmolyte and low-temperature clearly differed among the six proteins when probed by CD spectroscopy. However, these differences among the proteins were not uniform under the different conditions and were not related to the two sequence clades. Likewise, we found no correlation of the extent of folding with any of the physicochemical characteristics of the proteins. Above 8 osM, α-helix stabilization was much more pronounced for glycerol than for EG for COR15A and LEA25. This was not true for the remaining LEA proteins. COR15A oligomerizes in response to high glycerol concentrations [33]. Whether such oligomerization also occurs for LEA25 and whether this is related to the increased folding, which is actually higher than the maximum folding potential of the monomers in TFE, remains to be determined. 

Complete desiccation of the six LEA proteins led to the formation of mainly α-helical structure with no indication of aggregate formation. Rehydration at low RH induced partial aggregation, apparent as a shoulder in the Amide I peak at around 1620 cm^−1^. This was fully reversible upon further increasing the water content in the samples by incubation above 75% RH. Only LEA40 retained traces of aggregates even after full rehydration. At very low RH, hydrophilic groups in amino acid side chains may not be fully saturated with water molecules, thus establishing intra- and/or intermolecular interactions leading to aggregation. It is, however, interesting that in most cases these aggregates were sufficiently unstable to dissolve upon further rehydration. Co-solutes such as sugars as alternative H-bonding donors or other potential binding partners may influence aggregation at low RH. For example, desiccation induced aggregate formation of LEA7 is reduced in the presence of isolated lactate dehydrogenase, a soluble leaf protein extract [35] or model membranes [44].

MD simulations monitoring the unfolding at different osmolyte concentrations have been instrumental to characterize the (partially) folded state of COR15A [31], suggesting a helix-loop-helix structure in agreement with experimental NMR data obtained in 20% TFE [39]. We used a similar homology modeling/MD simulation approach in 100% glycerol for the LEA proteins in this study. We included COR15A for comparison, but had to omit LEA25, due to its large size. In addition, we extended the simulation time from the previously 30 ns to 500 ns. For all proteins, we found fast partial unfolding in the first 30–50 ns. Unfolding simulations were in good agreement with experimental high osmolarity data for COR15A, LEA11 and LEA40, which are the smallest of the six LEA proteins. With increasing protein size, however, simulation results deviated from experimental data, yielding either underestimations (LEA29) or overestimations (LEA7) of α-helicity. Inspection of the structural models after 500 ns indicated that a common pattern among the LEA proteins was the sequential arrangement of short α-helical segments connected by flexible, unstructured linkers.

The function of LEA proteins and associated modes of action remain largely elusive. Several LEA proteins have been described to stabilize model membranes during freezing or drying [20,62]. For COR15A the stabilization of chloroplast membranes during freezing has been shown both *in vivo* and *in vitro* [19,63,64]. The ability of the COR15A clade proteins to stabilize model membranes resembling the lipid composition of inner chloroplast membranes during a freeze/thaw cycle varies [30]. Here, we have shown that this variability extends to all six LEA_4 proteins that were tested. Whether membrane stabilization is actually the physiological function of the proteins other than COR15A cannot be decided based of these *in vitro* experiments. However, all six LEA_4 proteins have the capability to stabilize ICMM membranes during freezing to different degrees. These differences were not related to sequence differences between the two LEA_4 clades. COR15A interacts with membranes via the hydrophobic face of its amphipathic α-helices [31] and this structural motif is shared among all six proteins. 

The importance of the 11-mer motif TAQAAKEKAXE was suggested from studies in *E. coli* cells expressing peptide variants with variable motif abundance [65,66]. The motif abundance is higher in the LEA7 clade (in total 17 times), compared to the COR15A clade (six times, as it is not present in LEA11 and COR15A), but is not related to membrane stabilization. Dure [28] suggested that this motif is involved in coiled coil formation. Our *in silico* predictions support this hypothesis, with a majority of motif stretches representing putative coiled coil domains. CD spectra of such domains are characterized by a θ_222_/θ_208_ ratio of >1, while single-stranded α-helices have θ_222_/θ_208_ ratios of ≤0.86 [67,68]. CD spectra representing the folded, mainly α-helical state of the six LEA proteins at high co-solute concentrations showed θ_222_/θ_208_ ratios of <0.9, thus not supporting the incidence of coiled-coils. Moreover, we did not find significant correlations between motif abundance, coiled coil propensity or hydropathy and the folding propensity, or the degree of membrane stabilization for the six experimentally characterized LEA_4 proteins. However, it is interesting to note that the θ_222_/θ_208_ ratio was >1 at least for LEA29, LEA40 and LEA25 in the dry state (Figure 7, [30]), suggesting that desiccation may induce super-helical structures. It is important to note that the capacity of membrane stabilization was not correlated with folding capacity in TFE or α-helix content at high osmolarity or low temperature. This is interesting, as we have recently shown that folding propensity is directly related to ICMM stabilization capacity in the case of COR15A and two COR15A mutants with more stable α-helical structure [39]. Apparently, this relationship does not hold across different proteins. 

Electrostatic interactions are essential in protein folding and in protein-membrane interaction. The LEA_4 proteins are characterized by high numbers of charged amino acids. With an almost perfect segregation of charged residues, expressed in the parameter κ and an even distribution of positively and negatively charged amino acids, they can be considered as Janus sequences or polyampholytes in the Das-Pappu phase diagram, largely free of long-range interactions between oppositely charged blocks [43]. Polyampholytes sample distinctly nonglobular conformations [43], in agreement with our SLS/DLS data. The LEA_7 clade proteins carry a slightly lower fraction of charged residues than the COR15A clade proteins. This was not related to folding or membrane stabilization capacity, because we found no significant correlations between membrane stabilization capacity or folding propensity with κ or the fraction of charged residues.

In summary, we report interesting new findings considering the complex structural plasticity of LEA proteins. In this context, transient and reversible β-sheet formation during dehydration as well as folding in response to low temperature should be emphasized. Our data indicate that the six experimentally characterized LEA_4 proteins have similar structural and functional characteristics despite their low sequence similarity. Nevertheless, they showed strong differences in their folding response to TFE, high solution osmolarity and low temperature, and their capacity to stabilize ICMM liposomes during a freeze/thaw cycle. Functionally and evolutionary, these differences might be related to their differential tissue or subcellular localization. Investigation of the two LEA_4 clades indicated no obvious relationships between sequence conservation, folding and function. In this context the surprisingly large differences in folding and function between LEA7 and LEA29 should be pointed out, because these two proteins share the highest sequence identity of 70% among the experimentally investigated proteins. This protein pair highlights the importance of including several LEA proteins in comparative analyses. When only comparing these two proteins, membrane stabilizing capacity was directly dependent on folding propensity and abundance of the 11-mer motif. The apparent lack of correlation among any of the investigated physicochemical parameters with the structural and functional characteristics suggests that specific spatial patterns, affecting for example surface charge and hydrophobicity, or the probability of local contacts in the conformational ensembles, are crucial for folding and function. To unravel these complex relationships will be a challenging task for future research in this field. 

## 4. Materials and Methods 

### 4.1. Bioinformatic Analysis

Multiple sequence alignment of all LEA_4 Pfam family proteins and calculation of the identity matrix were done using Clustal W 2.1 [69] with the default settings. The resulting dendrogram was drawn using the Wasabi software [70]. The FIMO tool [71] included in the MEME suite version 5.1.0 [72] was used for motif screening. Secondary structure and disorder predictions were done using Porter 5.0 [73] and CSpritz version 1.2 [74], respectively. Coiled coil prediction was done with Deepcoil [75]. CIDER and local CIDER [43,76] were used to calculate linear hydropathy, charge parameters (κ, NCPR, linear NCPR) and Das-Pappu phase diagrams, respectively. For all predictions, N-terminal signal sequences were removed, if present.

### 4.2. Modeling and Molecular Dynamics (MD) Simulations

Molecular models of COR15A, LEA7, LEA11, LEA29 and LEA40 were built on the I-TASSER server [77] using the mature amino acid sequences lacking the N-terminal signal sequences. MD simulations in 100% glycerol were performed using the Gromacs molecular dynamics simulation engine, versions 2018.4 and 2018.7 [78] and the OPLS-AA force field [79,80,81], closely following the recently published procedure for COR15A [31]. Briefly, the model of a folded protein in vacuum was centrally placed in a dodecahedron box with a minimum edge distance of 15 Å. Solvent box replicates were added by the tool solvate, implemented in Gromacs using a previously constructed solvent box [31] for 100% glycerol. Solution environment was electrostatically neutralized by six sodium ions using the genion tool. After energy minimization, the system was equilibrated by two 1 ns protein-solvent equilibration runs. In the first run, the number of particles, volume and temperature (NVT), in the second run, the number of particles, pressure and temperature (NPT) were fixed. To avoid premature unfolding, proteins were kept constrained during equilibration. Each MD simulation was carried out at 300 K for 500 ns in five repeats with time steps of 2 fs, periodic boundary conditions, 10 Å spherical cut-off for non-bonded interactions and a force-switching function of 10 Å for van der Waals terms [31]. For the Coulomb type PME was chosen with a 10 Å radius.

### 4.3. Cloning, Expression and Purification of Recombinant LEA Proteins 

The respective procedures for COR15A, LEA11 and LEA25 were previously reported in detail [30,39]. The procedures for LEA7, LEA29 and LEA40 were analogous to that for COR15A reported in [39]. Briefly, all genes were amplified from RIKEN full-length cDNA clones [82,83] to encode the mature proteins lacking the N-terminal signal peptides [24], if present. All genes were cloned into expression vectors suitable for obtaining untagged recombinant protein either by tag-free expression (LEA11) or tag removal (COR15A, LEA7, LEA25, LEA29, LEA40). The identity of all inserts was validated by sequencing. Expression was done in the *E. coli* strains Rosetta or BL21 DE3. Cells were harvested by centrifugation and lysed by sonication. Heat soluble protein extracts were obtained by heat precipitation and subsequently purified by affinity or anion exchange chromatography as reported previously [30,39]. If present, affinity tags were removed by TEV protease cleavage followed by a second round of affinity chromatography for protease and HIS-tag removal. If necessary, a final purification step by size exclusion chromatography was applied, as previously reported for COR15A [39]. Protein solutions were either dialyzed against water, lyophilized and dissolved in the respective buffers or directly dialyzed against the respective buffers prior to measurement. Protein purity was validated by SDS-PAGE and Coomassie Blue staining, and dynamic light scattering. Protein concentrations were determined spectrophotometrically using the specific absorption at 280 nm calculated with the ProtParam tool on the ExPASy server [84]. 

### 4.4. Far-UV CD Spectroscopy

Far-UV CD spectra were recorded with a J-815 spectropolarimeter equipped with a Peltier-thermostat controlled cell holder (Jasco, Pfungstadt, Germany) using quartz cuvettes with appropriate path lengths of 0.1 or 1 mm (Hellma, Müllheim, Germany) in ddH_2_O or 10–20 mM NaH_2_PO_4_, pH 6.0 (LEA7, LEA40) or 7.4 (LEA29, COR15A) in the absence of co-solvent and with increasing concentrations of EG, glycerol or TFE. Spectra of dry proteins were measured from protein solutions dried on CaF_2_ windows overnight in vacuum. Protein concentrations of the dry samples were estimated from the absorbance at 193 nm measured parallel to the CD spectra in the spectropolarimeter as described previously [14]. Temperature curves were recorded at protein concentrations of 3 to 10 µM in 10 mM TES, 50 mM NaCl, 0.1 mM EDTA, pH 7.4 in 1 mm path length cuvettes at 222 nm between −5 °C and 95 °C with a constant heating rate of 1 °C/min in the absence or presence of 7 osM EG. Instrument calibration was done with 1S-(+)-10-camphorsulphonic acid. Estimation of α-helix ratio was done using θ_MRW_ at 222 nm [85]. 

### 4.5. FTIR Spectroscopy 

To expose proteins to different RH, protein solutions in ddH_2_O were spread on a CaF_2_ window and dried under vacuum for at least 2 h. Samples were then equilibrated in chambers of different defined RH at 20 °C for 24 h. RH were set to 97%, 93%, 85%, 75%, 33% and 11% using saturated solutions of K_2_SO_4_, KNO_3_, KCl, NaCl, MgCl_2_ and LiCl, respectively [86]. RH was continuously monitored using EL-USB-2 data loggers [34] (Lascar Electronics, Whiteparish, UK). Additionally, anhydrous samples (0% RH) and samples rehydrated over D_2_O (100% RH) were measured. Prior to measurement, samples were covered by a second CaF_2_ window to avoid rehydration. FTIR spectra were measured on a GX2000 FTIR spectrometer (PerkinElmer, Rodgau, Germany) and 32 spectra were accumulated and analyzed using the Spectrum 5.0.1 software (PerkinElmer, Rodgau, Germany). In addition, protein solutions in D_2_O were diluted with EG to generate a series of EG concentrations and 120 spectra were recorded and co-added using a 6 µm path length CaF_2_ Biocell (BioTools, Jupiter, FL, USA) in a Nicolet iS10 FTIR spectrometer (ThermoFisher, Dreieich, Germany).

### 4.6. Dynamic and Static Light Scattering (DLS/SLS) 

Simultaneous DLS/SLS experiments were done on a custom-built device equipped with a high quantum yield avalanche photo diode, a 0.5 W diode-pumped continuous-wave laser (Cobolt Samba 532 nm, Cobolt AB, Solna, Sweden) and an ALV 7002-25 ns correlator (ALV-GmbH, Langen, Germany) at 23 °C at a scattering angle of 90°, as described in detail previously [30,33]. All protein solutions were subjected to ultracentrifugation for 30 min at 60.000 g directly prior to measurement to remove air bubbles, dust and large aggregates. Protein concentrations were determined spectrophotometrically directly in the respective 3 mm-path length micro-fluorescence cuvettes (105.251-QS, Hellma, Müllheim, Germany). Viscosities and refractive indices of the used buffers were determined using an Ubbelohde-type viscometer (Viscoboy-2, Lauda, Königshofen, Germany) and a refractometer at 23 °C, respectively. 

### 4.7. Liposome Preparation and Carboxyfluorescein (CF) Leakage Assay 

Liposome stability during a freeze/thaw cycle in the absence and presence of LEA proteins was assessed as previously described [39]. Briefly, lipids purchased from Avanti Polar Lipids (Alabaster, AL, USA) were dissolved in chloroform, mixed in a glass tube and dried under nitrogen gas at 60 °C, followed by incubation under vacuum overnight to remove traces of solvent. Dry lipids were hydrated in 100 mM CF (Molecular Probes, Eugene, OR, USA), 10 mM TES, 0.1 mM EDTA, pH 7.4 and large unilamellar vesicles were formed by extrusion through two polycarbonate membranes with 100 nm pore size (Nucleopore, GE Healthcare, Freiburg, Germany) in a handheld extruder (Avanti Polar Lipids, Alabaster, AL, USA). Liposomes were separated from free CF by size exclusion chromatography, using a S75 13/300 size exclusion column equilibrated and eluted with 10 mM TES, 50 mM NaCl, 0.1 mM EDTA (pH 7.4) connected to an FPLC ÄKTA system (GE Healthcare, Freiburg, Germany), utilizing the absorption of CF at 280 nm. Liposomes were mixed with protein solutions in the same buffer at the indicated mass ratios, resulting in a final liposome concentration corresponding to about 6 mM lipid. Samples were rapidly frozen in an EG bath precooled to −20 °C, incubated at that temperature for 2 h and subsequently thawed at room temperature. CF leakage was determined with a Viroskan flash plate reader (Thermo Scientific, Waltham, MA, USA) using an excitation wavelength of 492 nm and an emission wavelength of 517 nm before and after disrupting the liposomes with Triton X-100 (Merck, Darmstadt, Germany) in 96-well fluorescence plates. CF leakage from the liposomes was calculated as described previously [39].

### 4.8. Correlation Analysis

Selected data sets were correlated using Pearson product moment correlation analysis. Correlation coefficients and the corresponding *p* values are summarized in Table S3.

## Supplementary Materials

Supplementary materials can be found at https://www.mdpi.com/1422-0067/21/8/2794/s1.

## Abbreviations

CD	Circular dichroism
CF	Carboxyfluorescein
COR	Cold regulated
DGDG	Digalactosyldiacylglycerol
DLS/SLS	Dynamic and static light scattering
EG	Ethylene glycol
EPG	L-α-phosphatidylglycerol (Egg, Chicken)
FCR	Fraction of charged residues
FTIR	Fourier-transform Infrared
ICMM	Inner chloroplast membrane modeling
IDP	Intrinsically disordered protein
LEA	Late embryogenesis abundant
MD	Molecular dynamics
MGDG	Monogalactosyldiacylglycerol
NCPR	Net charge per residue
NMR	Nuclear magnetic resonance spectroscopy
Pfam	Protein families
POPC	1-palmitoyl-2-oleoyl-phosphatidylcholine
RH	Relative humidity
R_S_	Hydrodynamic radius
SD	Standard deviation
SQDG	Sulfoquinovosyldiacylglycerol
TFE	2,2,2-trifluoroethanol
