# Supplementary material for: Similar Yet Different–Structural and Functional Diversity among Arabidopsis thaliana LEA_4 Proteins
Source: Int J Mol Sci. 2020 Apr 17;21(8):2794. doi: 10.3390/ijms21082794 (PMC7215670; doi:10.3390/ijms21082794)
Supplement: Supplementary file 1 [file ijms-21-02794-s001.pdf]

Supplemental Table 1: AGI codes, expression and subcellular localization of all 18 members of the LEA_4 Pfam family. Expression data are from [22], subcellular localization data from [23]. Experimentally characterized proteins are indicated with bold letters in blue (LEA7 clade) or red (COR15A clade). Sequential arrangement follows the one from Fig. 1. Amino acids and molecular mass are given for the mature proteins lacking signal peptides if present.

LEA protein	AGI code	Expression	Subcellular localization	Molecular mass [Da]	Amino acids
**LEA7**	**At1g52690**	**Bud, seed and stress**	**Cytosol and nucleus**	**18101**	**169**
**LEA29**	**At3g15670**	**Seed**	**Cytosol and nucleus**	**24186**	**225**
**LEA40**	**At4g13560**	**Reproductive tissues**	**Cytosol and nucleus**	**11584**	**109**
LEA28	At3g02480	Reproductive, seed and salt	Cytosol and nucleus	7145	68
LEA13	At2g18340	Seed	ER	49838	456
LEA43	At4g36600	Seed	ER	36438	335
LEA30	At3g17520	Seed	ER	32559	298
LEA42	At4g21020	Seed	Chloroplast and mitochondria	25304	226
LEA48	At5g44310	Seed	Chloroplast and mitochondria	33980	301
LEA9	At1g72100	Seed	Pexophagosome	52704	480
LEA39	At4g13230	Bud	Cytosol and nucleus	13064	120
LEA19	At2g36640	Seed		48493	448
LEA36	At3g53040	Seed	Cytosol and nucleus	52084	479
**LEA11**	**At2g03740**	**Bud**	**Chloroplast**	**15036**	**139**
LEA12	At2g03850	Bud	Chloroplast	14972	137
**LEA25**	**At2g42560**	**Seed and salt**	**Cytosol**	**67195**	**635**
COR15B	At2g42530	Non-seed and stress	Chloroplast	9728	90
**COR15A**	**At2g42540**	**Non-seed and stress**	**Chloroplast**	**9424**	**90**

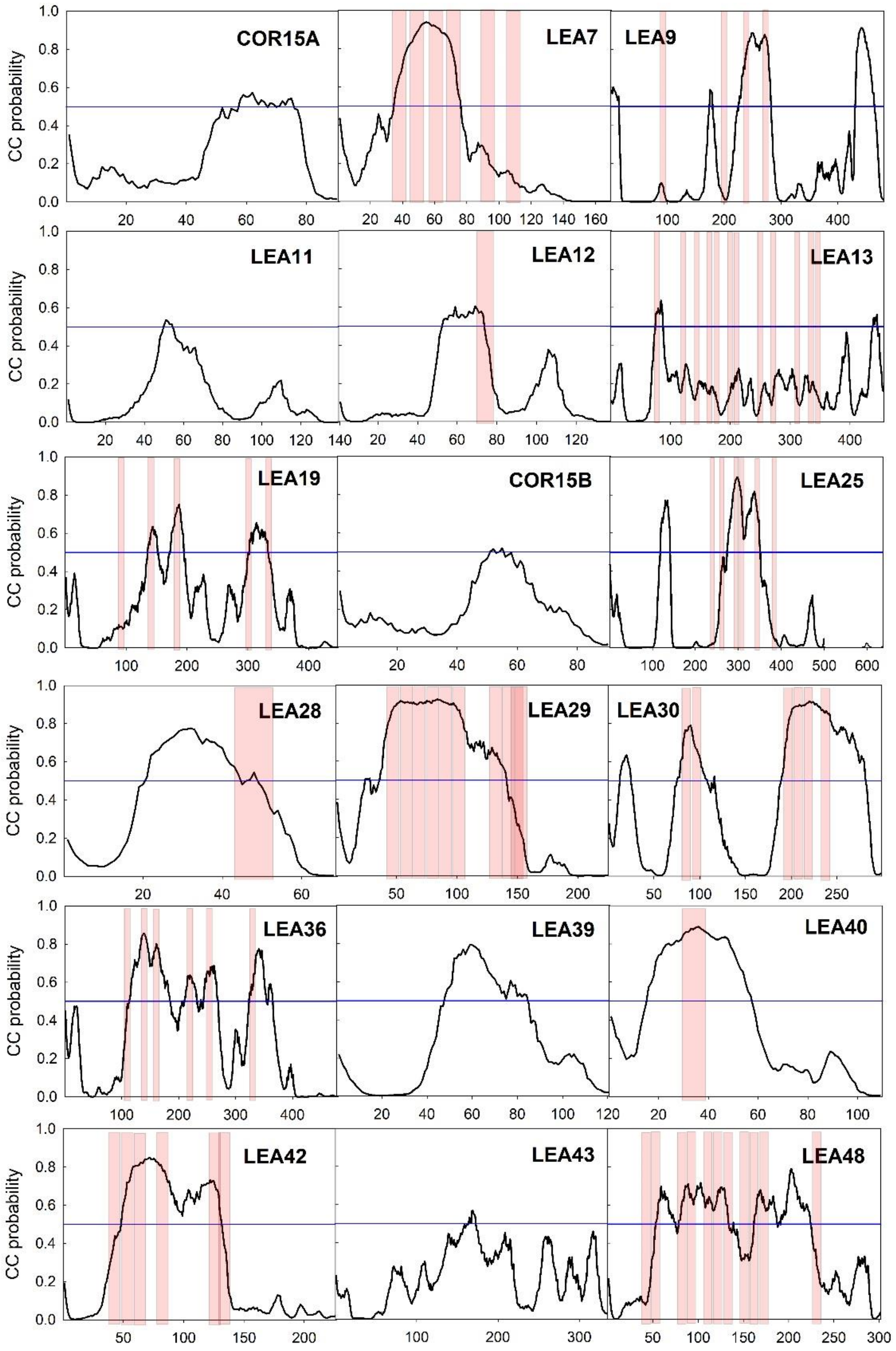

Supplemental Figure 1: Prediction of coiled coils for all *A. thaliana* LEA_4 protein sequences using Deepcoil. The x-axis depicts amino acid position. The blue line depicts the decision threshold for coiled coil formation. Red bars indicate sequence positions of the 11-mer motif.

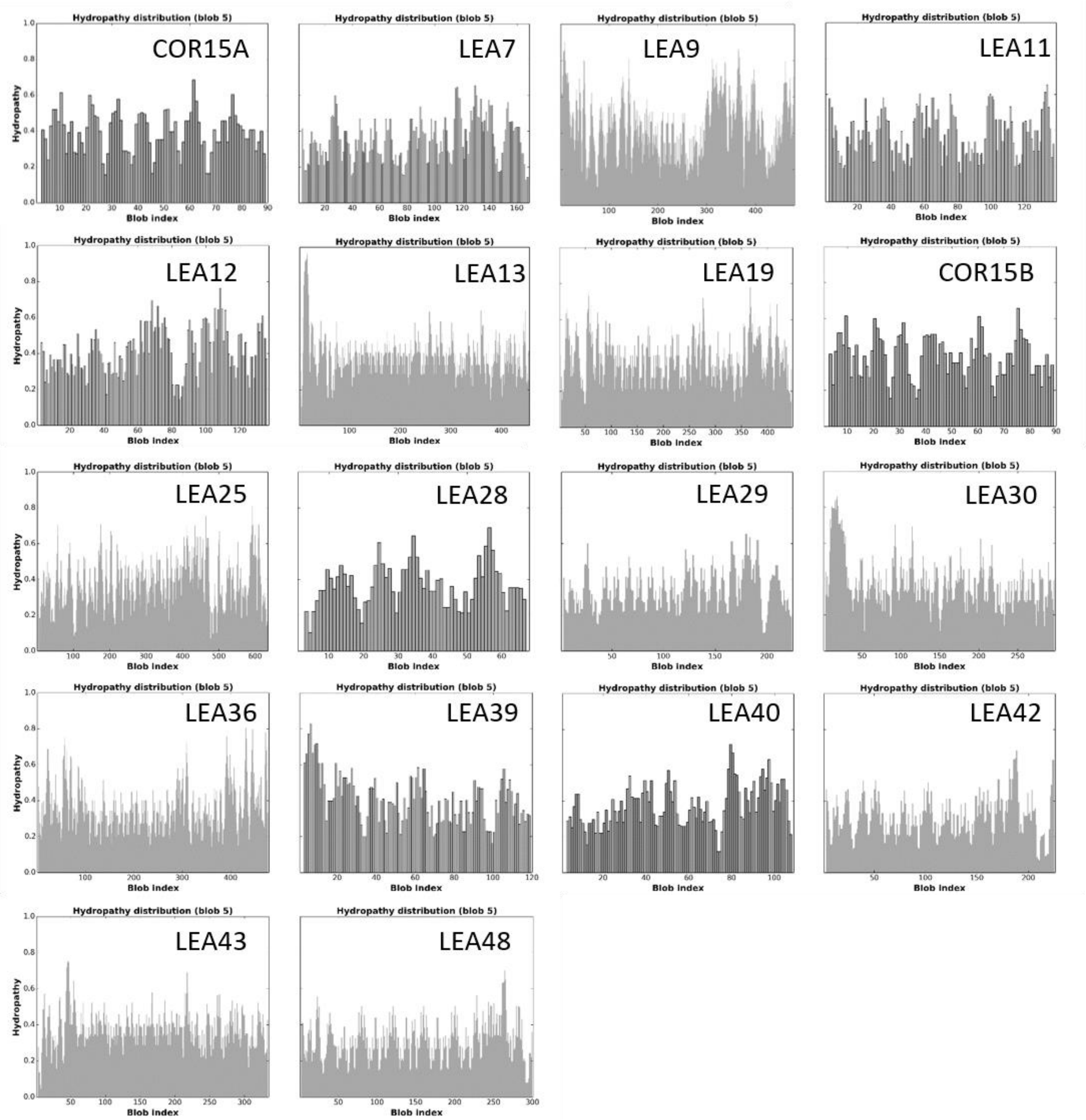

Supplemental Figure 2: Linear hydropathy plots for all *A. thaliana* LEA_4 protein sequences using CIDER. Hydropathy was calculated using a sliding window of five.

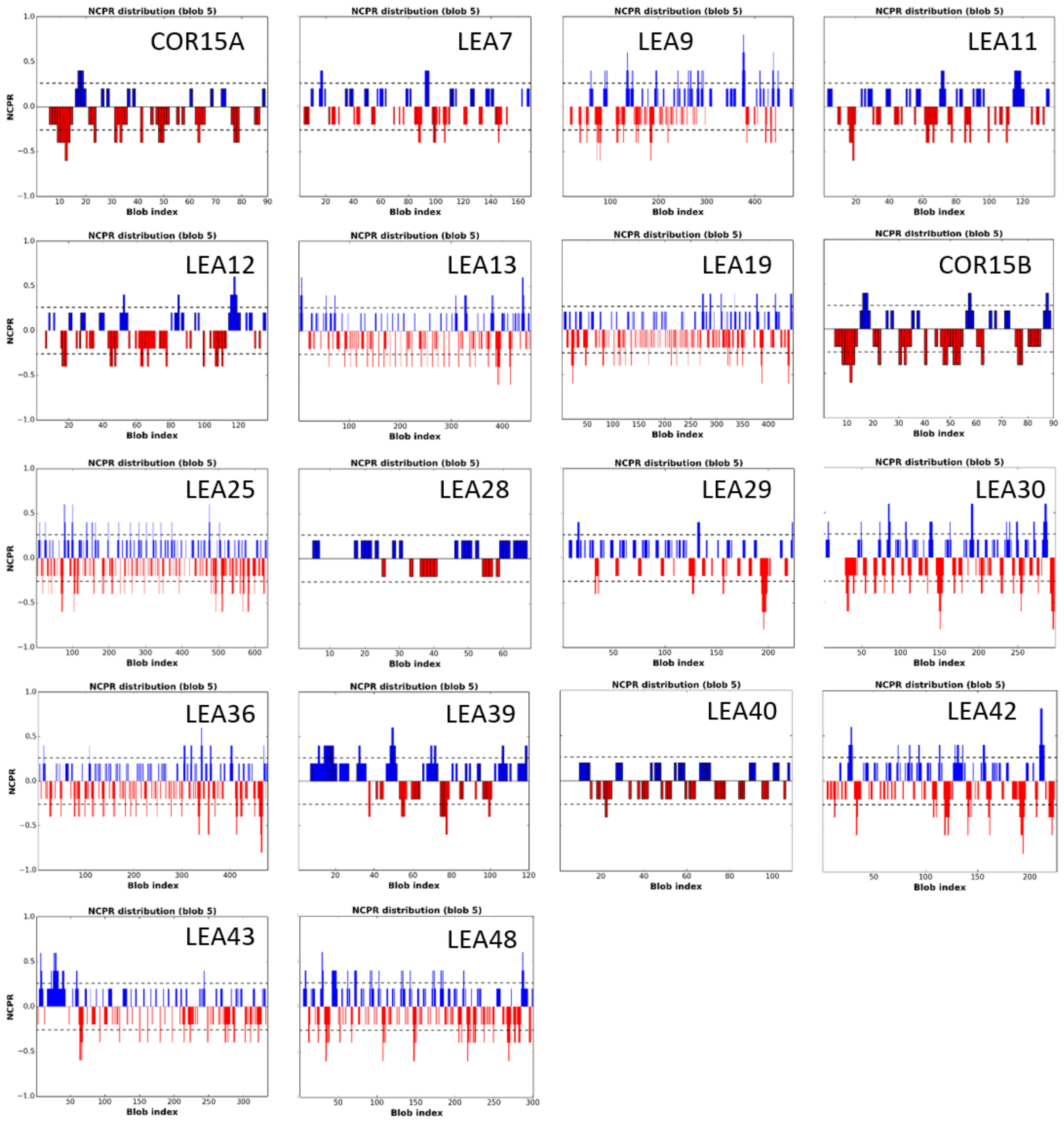

Supplemental Figure 3: Linear NCPR (net charge per residue) plots for all *A. thaliana* LEA_4 protein sequences using CIDER. NCPR was calculated using a sliding window of five.

Supplemental Table 2: Charge parameters for all *A. thaliana* LEA_4 protein sequences calculated using CIDER. The table contains fractions of negatively (f-) and positively (f+) charged residues, fraction of charged residues (FCR), net charge per residue (NCPR) and charge segregation along the sequence (κ) for each protein sequence and the average and standard deviation over all 18 sequences.

	**f-**	**f+**	**FCR**	**NCPR**	**κ**
**LEA7**	0.15	0.15	0.30	0.00	0.07
**LEA29**	0.13	0.15	0.28	0.01	0.08
**LEA40**	0.12	0.12	0.24	0.00	0.08
**LEA28**	0.12	0.15	0.26	0.03	0.05
**LEA13**	0.19	0.17	0.36	-0.02	0.07
**LEA43**	0.17	0.17	0.35	0.00	0.09
**LEA30**	0.22	0.18	0.40	-0.03	0.13
**LEA42**	0.25	0.23	0.47	-0.02	0.08
**LEA48**	0.26	0.25	0.50	-0.01	0.06
**LEA9**	0.14	0.14	0.28	0.01	0.11
**LEA39**	0.14	0.21	0.35	0.07	0.11
**LEA19**	0.21	0.19	0.40	-0.02	0.05
**LEA36**	0.23	0.20	0.43	-0.03	0.07
**LEA11**	0.19	0.18	0.37	-0.01	0.07
**LEA12**	0.20	0.18	0.37	-0.02	0.09
**LEA25**	0.18	0.15	0.33	-0.03	0.10
**COR15B**	0.24	0.18	0.42	-0.07	0.08
**COR15A**	0.24	0.18	0.42	-0.07	0.08
**Average**	0.18	0.18	0.36	-0.01	0.08
**Standard deviation**	0.05	0.03	0.07	0.03	0.02

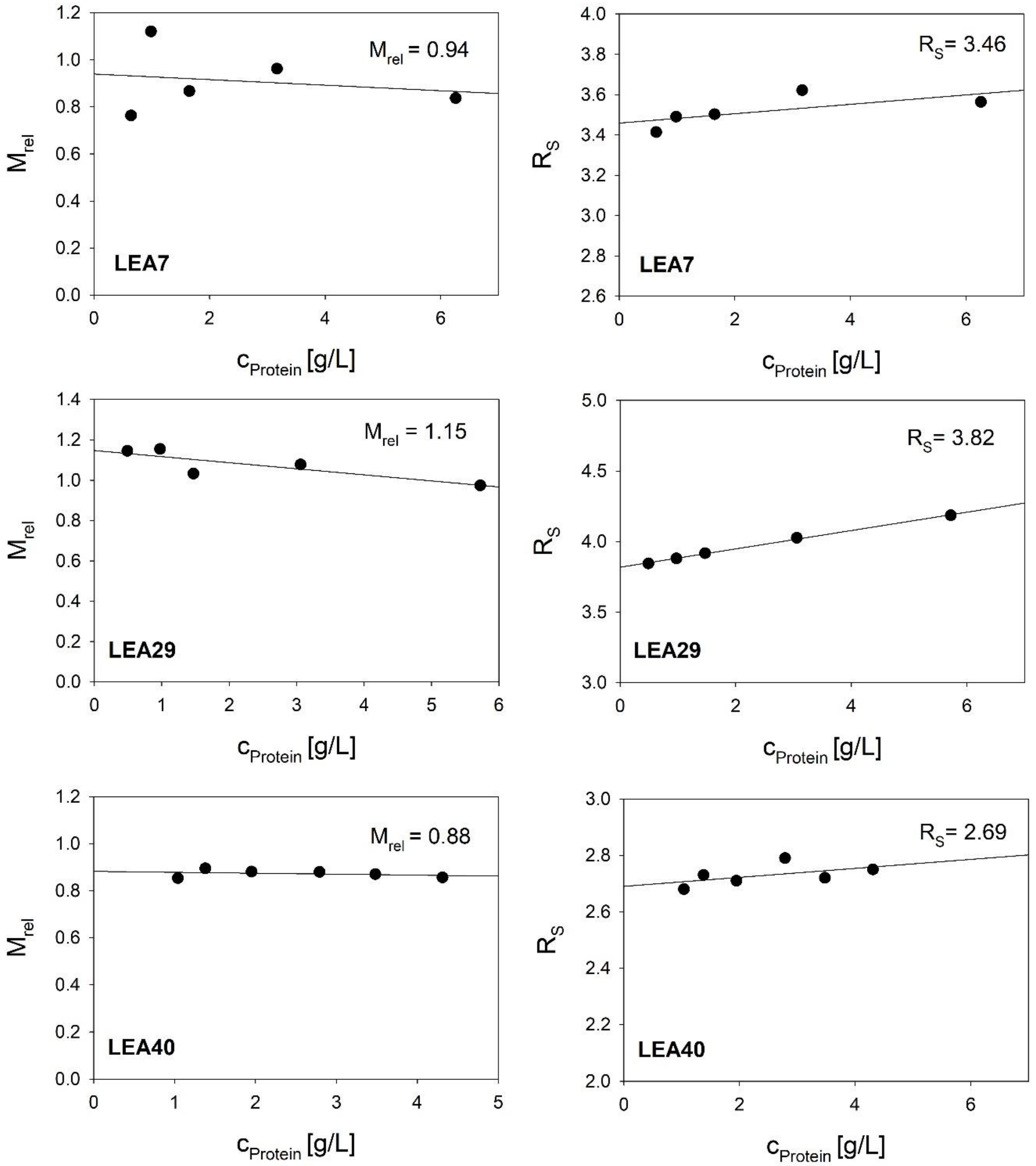

Supplemental Figure 4: Apparent relative masses (M_{rel} = apparent mass/theoretical mass of the monomer) (left panels) and R_S (right panels) dependent on the protein concentration for LEA7, LEA29 and LEA40. R_S and M_{rel} after extrapolation to infinite dilution are indicated.

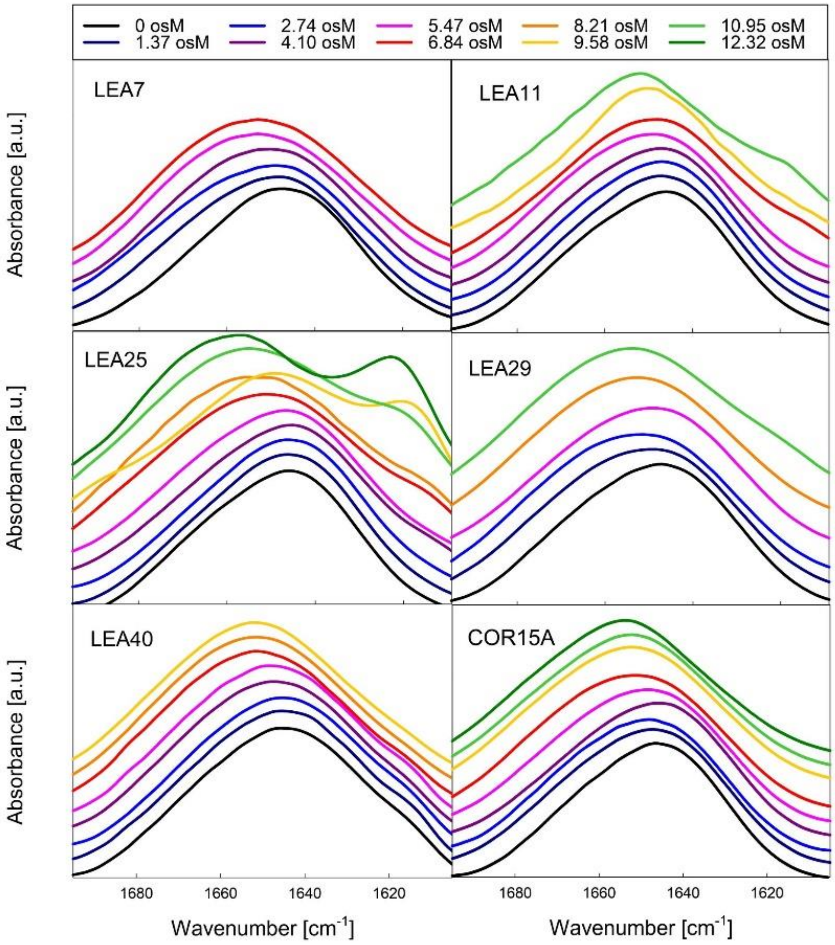

Supplemental Figure 5: Amide I peak from FTIR spectra of the six LEA_4 family proteins in an osmolarity gradient using ethylene glycol. Peak maxima indicate unstructured proteins (1640-1650 cm-1), α-helix (1660-1650 cm-1) and β-sheet aggregates (about 1620 cm-1). The peaks in each panel are offset from each other for better visibility.

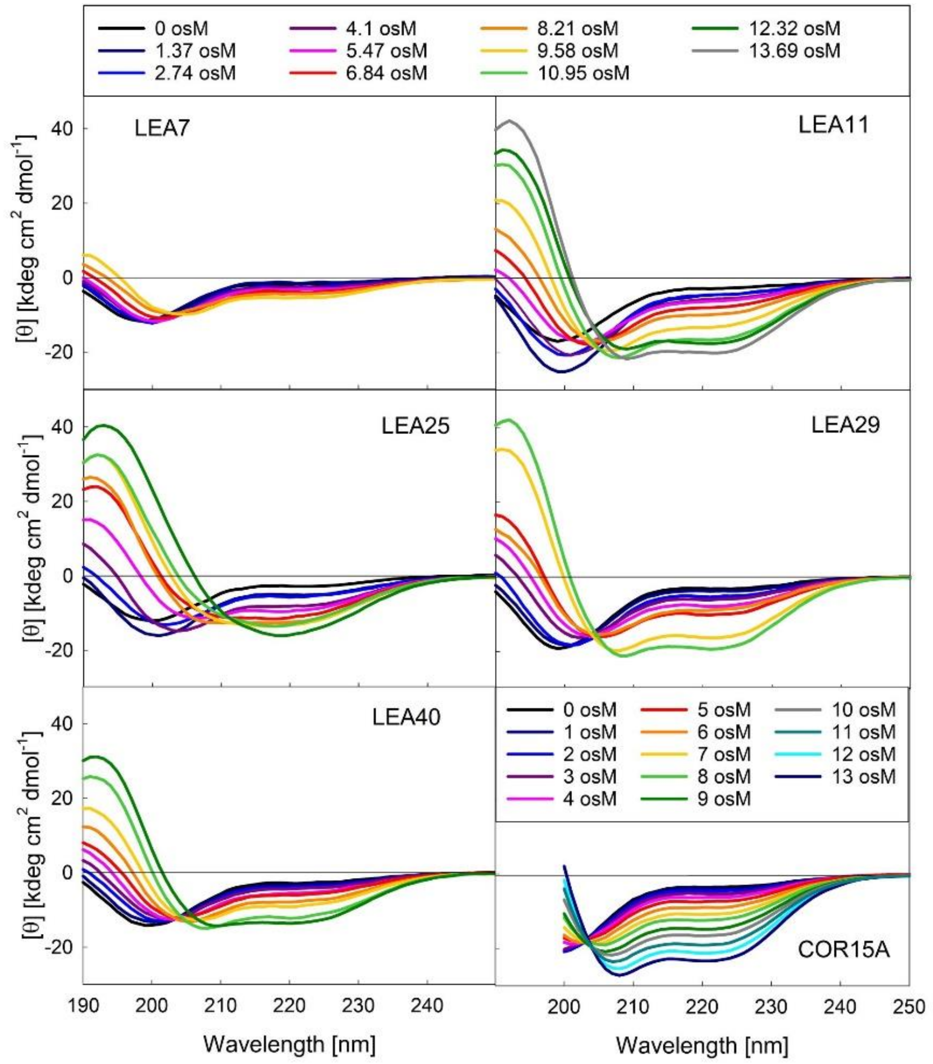

Supplemental Figure 6: Far-UV CD spectra of the six LEA_4 family proteins in increasing concentrations of the osmolyte ethylene glycol indicate folding. The legend in the figure head applies for LEA7, LEA11, LEA25, LA29 and LEA40, the legend in the lower right subpanel applies for COR15A.

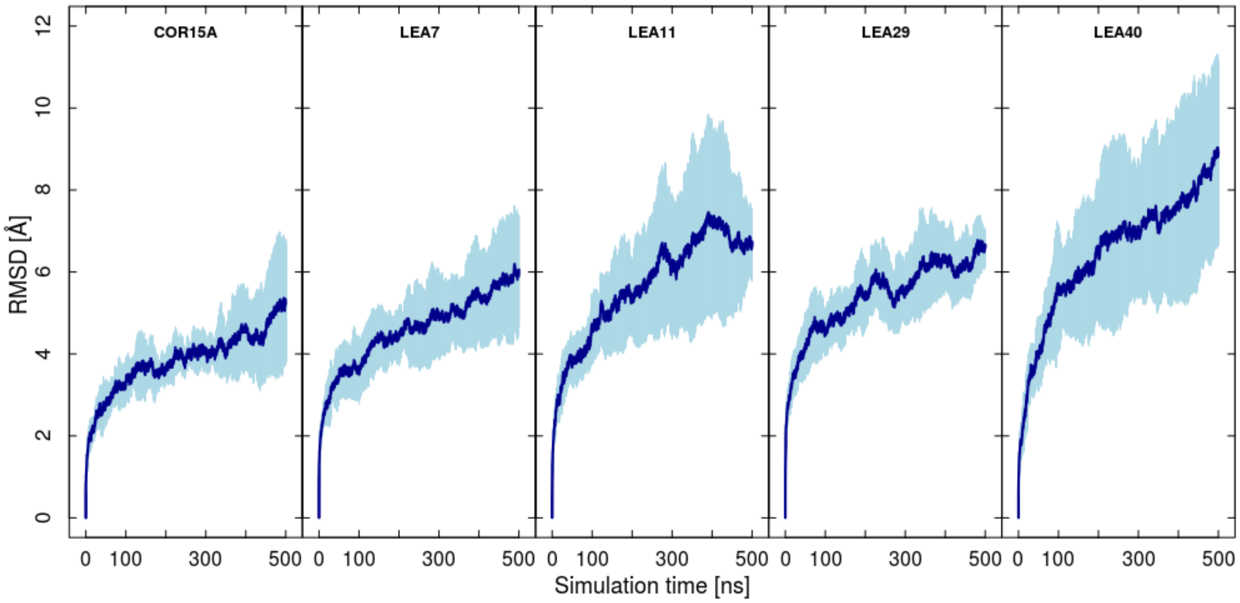

Supplemental Figure 7: RMSD during 500 ns MD simulations in 100 % glycerol. Data represent averages from five simulation replicates with error bars indicating the standard deviation.

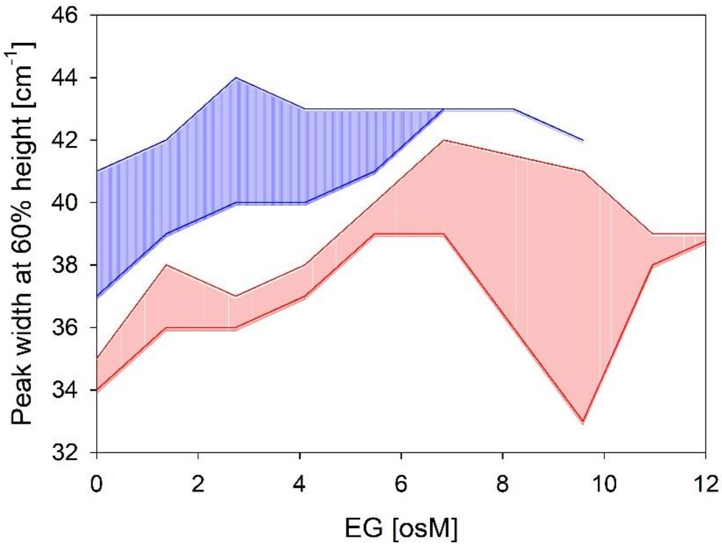

Supplemental Figure 8: Variance in the FTIR Amid I peak width at 60% height of the two LEA_4 clades as a function of EG concentration. Highest and lowest peak width within the LEA7 clade (blue) and the COR15A clade (red) represent the limits of the colored areas.

Supplemental table 3: Pearson correlation coefficients and p-values of selected traits of the six experimentally characterized LEA proteins. CF leakage data from 1:100 protein: lipid mass ratio were used. Correlation coefficients with $p < 0.05$ are shown in bold.

	Θ_{222} nm in 10 osM EG	Θ_{222} nm at -5 °C in 7 osM EG	CF leakage	Abun-dance of 11-mer motif	Hydro-pathy	κ	FCR	Coiled coil [%]
Θ_{222} nm in 30% TFE	**0.863**<br>**0.0270**	0.299<br>0.565	0.139<br>0.792	0.205<br>0.697	-0.283<br>0.587	0.0568<br>0.915	-0.625<br>0.184	0.0789<br>0.882
Θ_{222} nm in 10 osM EG		0.463<br>0.355	0.259<br>0.620	-0.0249<br>0.963	-0.374<br>0.465	-0.442<br>0.381	-0.523<br>0.287	-0.00460<br>0.993
Θ_{222} nm at -5 °C in 7 osM EG			-0.447<br>0.374	0.103<br>0.846	-0.416<br>0.412	-0.478<br>0.337	-0.438<br>0.386	-0.0501<br>0.925
CF leakage				-0.379<br>0.458	0.458<br>0.361	-0.140<br>0.791	0.593<br>0.214	-0.602<br>0.206
Abundance of 11-mer motif					-0.692<br>0.128	0.192<br>0.716	-0.466<br>0.351	0.591<br>0.217
Hydropathy						0.428<br>0.398	0.751<br>0.0853	-0.778<br>0.0683
κ							0.0328<br>0.951	0.0166<br>0.975
FCR								-0.736<br>0.0955
